# Interbreeding among deeply divergent mitochondrial lineages in the American cockroach (*Periplaneta americana*)

**DOI:** 10.1038/srep08297

**Published:** 2015-02-06

**Authors:** Christoph von Beeren, Mark Y. Stoeckle, Joyce Xia, Griffin Burke, Daniel J. C. Kronauer

**Affiliations:** 1 Laboratory of Insect Social Evolution, The Rockefeller University, New York, NY, USA; 2 Program for the Human Environment, The Rockefeller University, New York, NY, USA; 3 Hunter College High School, New York, NY, USA; 4 Bard College, Annandale-on-Hudson, NY, USA

## Abstract

DNA barcoding promises to be a useful tool to identify pest species assuming adequate representation of genetic variants in a reference library. Here we examined mitochondrial DNA barcodes in a global urban pest, the American cockroach (*Periplaneta americana*). Our sampling effort generated 284 cockroach specimens, most from New York City, plus 15 additional U.S. states and six other countries, enabling the first large-scale survey of *P. americana* barcode variation. *Periplaneta americana* barcode sequences (n = 247, including 24 GenBank records) formed a monophyletic lineage separate from other *Periplaneta* species. We found three distinct *P. americana* haplogroups with relatively small differences within (≤0.6%) and larger differences among groups (2.4%–4.7%). This could be interpreted as indicative of multiple cryptic species. However, nuclear DNA sequences (n = 77 specimens) revealed extensive gene flow among mitochondrial haplogroups, confirming a single species. This unusual genetic pattern likely reflects multiple introductions from genetically divergent source populations, followed by interbreeding in the invasive range. Our findings highlight the need for comprehensive reference databases in DNA barcoding studies, especially when dealing with invasive populations that might be derived from multiple genetically distinct source populations.

Globalization facilitates the introduction of invasive species that can damage native ecosystems, cause severe economic losses, and threaten human health^1^,^2^. Early detection and rapid response are cornerstones of successful management strategies. However, identification can be problematic, first and foremost due to a lack in taxonomic expertise^3^,^4^. DNA-based approaches such as ‘DNA barcoding' potentially provide relatively rapid and inexpensive species identifications^4^. Although the reliability and usefulness of DNA barcoding are subject of extensive debate^5^,^6^,^7^,^8^, this method has been shown to effectively distinguish species in many animal groups^9^,^10^,^11^,^12^,^13^. By comparing a short, standardized fragment of the mitochondrial gene *cytochrome c oxidase I* (*COI*) to a reference DNA barcode library, animal specimens can usually be assigned to species, as long as the database contains relevant reference sequences^14^,^15^. This approach has been applied to native populations of diverse animals^16^,^17^,^18^,^19^,^20^,^21^ and a variety of invasive taxa^3^,^22^,^23^,^24^,^25^. Successful invaders are generally presumed to represent expansions of a small founder population and are therefore likely genetically uniform^24^,^26^,^27^,^28^,^29^. For example, the invasive gall wasp *Quadrastichus erythrinae* shows a complete lack of mitochondrial as well as nuclear diversity across the Pacific, including Japan, Hawaii, Guam, and Samoa^30^, presumably reflecting a single outbreak starting with a small number of individuals. On the other hand, invasive populations may also represent multiple independent introductions, potentially from genetically distinct source populations^31^,^32^. Such a pattern of introduction may complicate the identification of invasive species via DNA barcodes, and may require more extensive sampling to establish a reliable reference library.

Here we assess DNA barcode variation in invasive populations of the American cockroach, *Periplaneta americana* (Linnaeus), one of the most abundant, widely distributed, and hated urban pests^33^,^34^. Though the native range of *P. americana* is unknown (possibly tropical Africa or South Asia^34^,^35^,^36^), all urban populations will be considered invasive in this study as it seems likely that they were established via human-aided dispersal. According to the World Health Organization, cockroaches are highly damaging pests worldwide in terms of potential health problems (allergies, asthma, and transmission of pathogens by contaminating food) and costs for pest control^37^. Furthermore, cockroaches are uniquely unpopular as most people find them disgusting, associating their presence with uncleanliness and disease^38^.

Although abundant and living in close proximity to humans, surprisingly little is known about *P. americana* biology outside the laboratory. Aspects of infestation control have been examined^39^,^40^,^41^,^42^, but few studies have investigated the biology of *P. americana* in urban settings^33^,^43^. Particularly strikingly, data on genetic variation is all but absent. The goal of this study was twofold: (1) to assess the genetic diversity of *P. americana* in urban populations, thus creating a valuable reference DNA barcode library; (2) to test the potential of DNA barcoding for quick and accurate molecular identification of this urban pest species. To facilitate specimen collection, we set up a citizen science project based in New York City (NYC), a center of world commerce and home to an abundance of cockroaches as well as more than eight million people^44^,^45^,^46^. We received several hundred specimens from scores of locations in NYC and beyond, enabling the first large-scale study of genetic diversity and providing a reference DNA barcode library for this important urban pest. We found deeply divergent mitochondrial lineages that could naively be interpreted as indicative of multiple cryptic species. However, nuclear DNA sequence data revealed extensive gene flow between mitochondrial clades, consistent with a single biological species. Our findings highlight the value of expanded sampling to accurately delineate species boundaries via DNA barcoding, in particular for invasive species.

## Results

**Figure 1:**
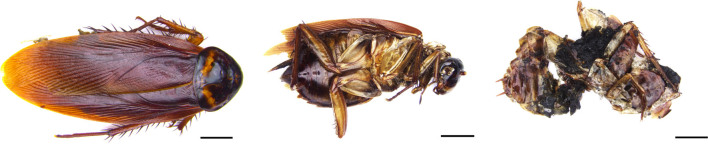
Representative *P. americana* specimens, ranging from well preserved (left) to substantially damaged (right). Scale bars are 5 mm.

The collection effort of 85 participants generated 284 specimens from 16 U.S. states and Argentina, Australia, Belize, Guyana, Spain, and Venezuela (Supplementary Tab. S1). In addition, 24 *P. americana* *COI* GenBank records were included in genetic analyses representing samples from Iran, China, and Korea (Supplementary Tab. S2). Specimen conditions ranged from well preserved to substantially damaged (Fig. 1). *COI* barcodes were successfully recovered from 238 cockroach specimens (including 223 *P. americana* specimens), while either PCR or sequencing failed repeatedly for 46 specimens (Supplementary Tab. S1). Seven sequences were trimmed at one end (range: 3–30 base pairs (bp)) due to low quality signals. The remainder were high-quality, full-length reads of 658 bp (N = 231) that contained 43 variable and parsimony-informative sites, as well as three singletons. Among the failed 46 specimens were eight specimens morphologically identified as the German cockroach (*Blatella germanica*). These specimens yielded a short PCR product (approximately 400 bp). Sequencing of one of these short PCR products revealed a putative nuclear pseudogene with a 285 bp internal deletion and multiple amino acid substitutions compared to available *B. germanica* GenBank records, including a complete mitochondrial genome (GenBank EU854321). This aberrant sequence was identical to *B. germanica COI* GenBank records KC473901 and KC473904, which thus presumably represent the same pseudogene. Except for that short sequence, no stop codons, unusual amino acid substitutions, or internal sequence deletions were found in any other sequence, indicating that all other sequences were likely functional mitochondrial sequences and not nuclear pseudogenes. In addition to *COI* barcodes, we analyzed a portion of the nuclear gene *wingless* (*wg*) for a subset of specimens. All *wg* amplifications yielded a full length, high quality sequence (N = 80; 77 *P. americana* and three *P. fuliginosa* specimens). The *wg* alignment for *P. americana* contained 8 variable and parsimony-informative sites, as well as one singleton.

**Figure 2:**
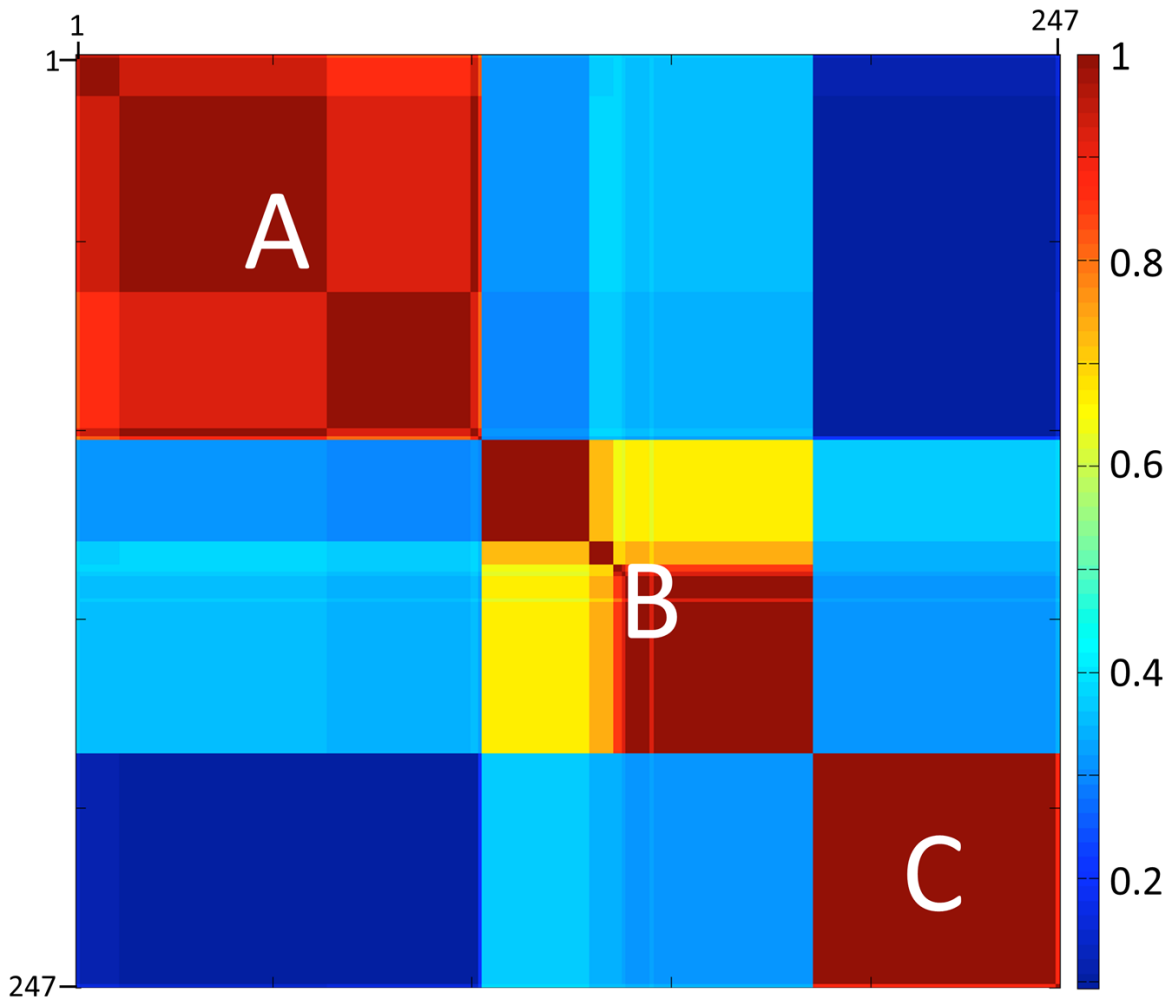
Klee diagram of *COI* barcodes. A Klee diagram is a colorized matrix of indicator vector correlations; identical sequences have a correlation of 1^87^. The dataset comprises 247 *P. americana* sequences (including 24 from GenBank). The matrix is arranged according to a NJ tree. Labeled blocks of high correlation along the diagonal correspond to *P. americana* haplogroups A, B, and C. The 15 individual haplotypes appear as sub-boxes. The color code for correlation coefficients is given on the right.

**Figure 3:**
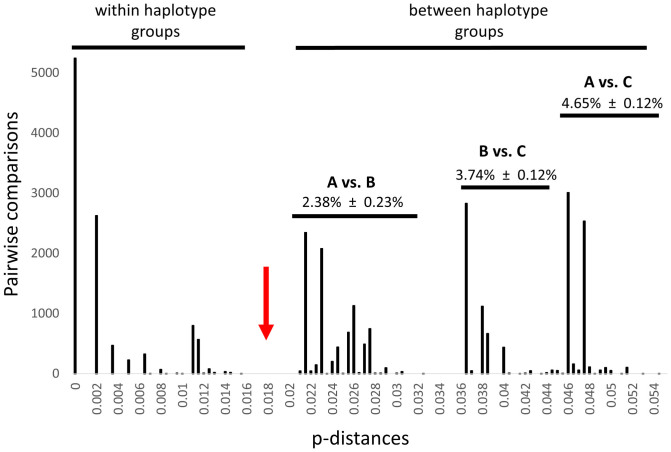
Histogram showing intra- and inter-haplogroup p-distances between *P. americana* mitochondrial *COI* sequences (N = 247). An apparent barcode gap (red arrow) separates maximum distances within and minimum distances between haplogroups. Mean ± SD p-distances between groups are shown.

The American cockroach showed an unusual pattern of genetic diversity with three major mitochondrial haplogroups (A, B, C) (Figs. 2, 3). Including the 24 GenBank sequences, the three groups comprised 15 haplotypes, six of which were represented in NYC (Supplementary Tables S1, S2). Mean p-distances between groups were larger (ranging from 2.38% to 4.65%) than within groups (mean p-distance ± SD, A: 0.12% ± 0.12%, B: 0.57% ± 0.52%, C: 0.03% ± 0.14%; N = 247 specimens) (Fig. 3). Among NYC specimens, p-distances were very similar to those in the complete dataset (mean p-distances between groups: 2.34%–4.63%; variation within groups, A: 0.10% ± 0.09%, B: 0.44% ± 0.52%, C: 0.00% ± 0%; N = 161 specimens). Thus, apparent ‘barcoding gaps' existed between maximum intra-haplogroup and minimum inter-haplogroup p-distances (Fig. 3).

**Figure 4:**
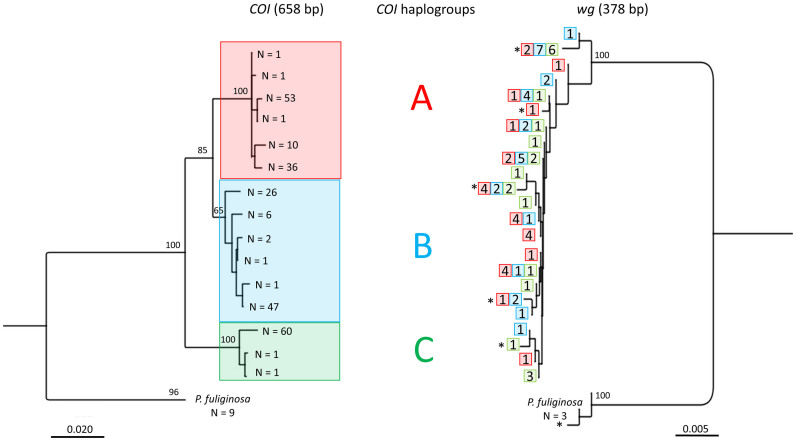
Lack of congruence between mitochondrial and nuclear data. Comparison of NJ trees based on Tamura-Nei distances for mitochondrial (*COI*, N = 256) and nuclear (*wg*, N = 80) DNA sequences of *P. americana* and *P. fuliginosa*. Colors depict different *COI* haplogroups. Numbers on the *wg* tree give the sample sizes (number of individuals) and boxes in one row represent individuals with the same allele combination. *Periplaneta fuliginosa* was used as outgroup in both analyses. Bootstrap support for major nodes is shown. Abbreviations: N = number of individuals, * = homozygous individuals.

**Figure 5:**
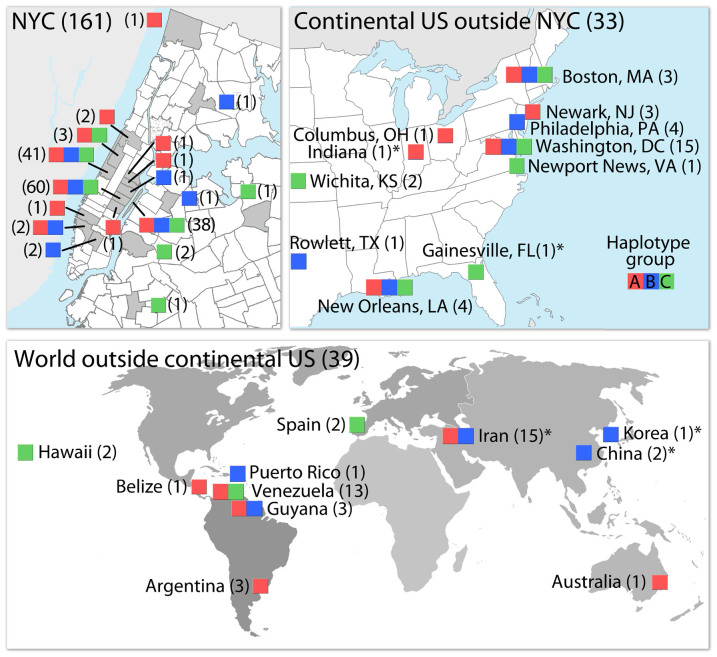
Distribution of *P. americana* *COI* haplogroups in NYC, continental U.S., and the world. Sample sizes per site are given in parentheses, haplogroup colors and designations correspond to those in Figure 4. NYC zip codes and U.S. states are outlined on the maps in the upper left and upper right, respectively. Sequences retrieved from GenBank are marked with asterisks. Maps were created with Adobe Photoshop. Map templates are from d-maps.com (US, http://d-maps.com/carte.php?num_car=5222&lang=en; world, http://d-maps.com/carte.php?num_car=3267&lang=en) and U.S. Census Bureau (NYC zip codes, https://www.census.gov/geo/maps-data/data/cbf/cbf_zcta.html).

Mitochondrial (*COI*) and nuclear (*wg*) phylogenetic trees were highly discordant, i.e., nuclear gene analysis did not recover the three major clades (Fig. 4). Specimens from different *COI* haplogroups shared the same *wg* alleles, and specimens from the same haplogroup carried different *wg* alleles. Overall, we found no genetic differentiation at the *wg* locus among *COI* haplogroups, indicating interbreeding (within NYC population: overall F_ST_ = 0.041, P = 0.100, individuals: N_haplogroup A_ = 20, N_haplogroup B_ = 26, N_haplogroup C_ = 14, alleles: N_haplogroup A_ = 7, N_haplogroup B_ = 6, N_haplogroup C_ = 6; pairwise comparisons: haplogroup A vs. B: F_ST_ = 0.069, P = 0.053; haplogroup A vs. C: F_ST_ = 0.045, P = 0.13; haplogroup B vs. C: F_ST_ = −0.007, P = 0.462). The three haplogroups had broadly overlapping geographic distributions, including within NYC, across the U.S., and elsewhere in the world (Fig. 5).

All *P. americana* *COI* haplotypes formed a monophyletic lineage separate from other *Periplaneta* species obtained in this study or represented in GenBank (Supplementary Fig. S1). The closest p-distance was found with *Periplaneta australasiae* (minimum sequence divergence 7.4%). In addition to *P. americana*, four other cockroach species were identified by *COI* barcode in this study, comprising about 6% of our specimens, including Smokybrown cockroach (*P. fuliginosa*) (N = 9), Brown-banded cockroach (*Supella longipalpa*) (N = 2), Turkestan cockroach (*Shelfordella lateralis*) (N = 1), and Madagascar hissing cockroach (*Gromphadorhina portentosa*) (N = 1) (Supplementary Tab. S1). Finally, two specimens, one from the southern U.S. (Georgia) and one from Venezuela, could not be identified by barcode (Supplementary Tab. S1).

## Discussion

This is the first large-scale study of mitochondrial diversity in the American cockroach (*Periplaneta americana*)*.* Surprisingly we found three deeply-divergent, widely-distributed *P. americana* *COI* haplogroups. In a limited geographic area such as NYC, deeply divergent mtDNA lineages within a single species are unusual. Several scenarios can explain such a genetic pattern, which are not mutually exclusive: historical introgression between species^47^,^48^,^49^,^50^,^51^,^52^,^53^, manipulation by endosymbiotic bacteria^54^,^55^, secondary contact of formerly isolated populations^56^,^57^, or reproductive barriers among sympatric cryptic species^17^,^19^. The latter scenario does not apply in our case. The presence of a “barcode gap” between the maximum distances within and minimum distances between haplogroups has often been proposed to indicate cryptic species in native populations^7^,^16^,^18^,^19^,^58^,^59^ (however, for critical views see refs. 8, 55, 60, 61). Although we observed clear barcode gaps in *P. americana*, the nuclear data are indicative of a single biological species. Cryptic speciation should be reflected in a detectable differentiation at nuclear markers, in particular in such deeply divergent mtDNA clades (up to 4.6% sequence divergence). This was not the case, and our analysis of nuclear sequences revealed extensive gene flow among *COI* haplogroups.

Discordant phylogenetic signals between maternally and biparentally inherited markers are sometimes due to infection with endosymbiotic bacteria^54^,^55^. *Wolbachia* bacteria, for example, transmit maternally and manipulate host reproduction in favor of infected females^62^,^63^. As mitochondria are maternally transmitted as well, selection favors those mtDNA types that are associated with *Wolbachia* infections, which can create unexpected mitochondrial population structures^55^. Vaishampayan and colleagues^64^ detected *Wolbachia* infections in cockroaches of the genera *Blattella* and *Supella*, but not in *P. americana*, suggesting that *Wolbachia* infections in *P. americana* might be rare or absent. In general, *Wolbachia* infections will substantially reduce mtDNA diversity in a given population and skew the frequency distribution of alleles towards a single or very few variants (the latter in cases of multiple infections)^55^,^65^,^66^. The pattern of mtDNA diversity detected in this study does, however, not reflect the typical pattern of reduced haplotype diversity found in *Wolbachia*-infected populations. We detected six different, divergent haplotypes in a single population of *P. americana* in NYC, and this general pattern seems to hold for other populations around the globe. Thus, it seems unlikely that *Wolbachia* infections have played a major role in creating the unusual pattern of mtDNA variation detected in this study*.* However, additional work screening specifically for *Wolbachia* infections will be required to conclusively rule out this possibility. Likewise, there is currently no evidence for historical introgression from other *Periplaneta* species. All available *P. americana* *COI* barcodes formed a monophyletic lineage clearly separated from congeneric species (Supplementary Fig. S1). Future studies may, however, uncover overlap in mitochondrial haplotypes with other species not yet represented in databases.

Currently, the most likely explanation for the detected genetic pattern is multiple human-mediated introductions from allopatric source populations followed by global dispersal among commercial centers. In fact, the different haplogroups must have diverged long before human-aided dispersal, even if the highest mutation rate estimates of insect mtDNA are applied (10–20% sequence divergence per million years; see Papadopoulou et al. 2010 for a review on mtDNA clocks in insects^67^). *Periplaneta americana*'s ability to inhabit human-built structures^33^,^34^ has probably facilitated its introduction to new areas. In general, human-mediated transport creates many opportunities for introduction and interbreeding of previously isolated species or populations^1^,^2^. For example, Ruddy Ducks artificially introduced to the UK from North America hybridize freely with the indigenous White-headed Duck, effectively threatening extinction of the native form^68^. In the present case, it appears that *P. americana* individuals from three or more historically isolated geographic populations are now effectively merged into a single global gene pool.

Invasive species often represent multiple introductions from genetically distinct source populations, and interbreeding may both be common and critical for long-term invasion success^1^,^26^,^31^,^32^,^69^,^70^,^71^,^72^. For example, interbreeding between distinct genetic lineages in the multicolored ladybird beetle (*Harmonia axyridis)* led to shifts in key life history traits enabling invasion success^73^,^74^. Conversely, invasive populations are often derived from small founding populations, and this genetic bottleneck can entail a substantial reduction in genetic diversity and lead to inbreeding depression^1^. The success of invasive species in their non-native range might be temporary in many cases, because prolonged inbreeding generally leads to a decrease in fitness^75^,^76^,^77^ and, possibly, population extinctions^78^,^79^. For example, inbreeding depression has been suggested as one mechanism underlying the collapse of New Zealand populations of one of the world's worst invasive pests, the Argentine ant (*Linepithema humile*)^80^. Empirical research on the influence of multiple introduction events on invasion success has just begun^1^, but examples suggest that preventing repeated introductions may reduce adaptive potential in some cases^32^ and thus may facilitate the long-term control of seemingly well-established invasive pests.

The spread of invasive species and thus of pest species like *P. americana* is expected to be facilitated and intensified by globalization. Our case study shows that species delimitation using a classical DNA barcoding approach can be particularly problematic when studying populations of invasive species, because existing populations might represent a mix of individuals from genetically distinct source populations. In such cases, it will be especially important to supplement mitochondrial DNA barcoding with nuclear genetic and/or morphological data. Nonetheless, with the comparatively large reference library provided in this study, DNA barcoding now has great potential to be employed as a quick and reliable tool to identify urban populations of the American cockroach.

## Methods

### Specimen collection and processing

To obtain specimens of the American cockroach, we set up a citizen science project based in New York City (NYC). The project was launched between November 2012 and January 2014 starting with a website including participant instructions (http://phe.rockefeller.edu/barcode/cockroachproject.html). We sought publicity via word of mouth and social media, as well as traditional outlets including television, newspapers, and radio. Contributors were asked to provide specimen collection date and location, and their name and contact information, and to send unpreserved dead specimens via regular mail.

Each specimen was assigned a code, transferred to an individual 50 mL Falcon vial, and stored at −30°C until further processing. Collection information was recorded in a spreadsheet (Supplementary Tab. S1). Six well-preserved *P. americana* specimens from each haplogroup (A, B, and C) were morphologically identified using the key of Helfer^81^. These samples are stored at the American Museum of Natural History (see Supplementary Tab. S1 for unique specimen identifiers). Other cockroach specimens were identified to species via DNA barcodes. *COI* barcode sequences were available in GenBank for most of the common urban roaches (see Supplementary Tab. S2 for reference sequences), allowing us to assign species names even to morphologically unrecognizable specimens.

### Genetic analysis

Genetic analysis was performed on 283 specimens (Supplementary Tab. S1). DNA was extracted from cockroach leg fragments using the QIAGEN® DNeasy® kit and stored at −30°C. The mitochondrial *COI* barcode region (658 base pairs) and a portion of the nuclear gene *wingless* (*wg*; 378 bp) were amplified in standard polymerase chain reactions (PCRs) using the primers LCO1490/HCO2198^82^ and wg550F^83^/wgcockR, respectively. The primer wgcockR (sequence: 5′ AACATGCACGCACACCTCTGCACCACGGACACC 3′) was designed specifically for this study, because longer fragments using primers wg550F/wgAbrZ^83^ did not amplify consistently. PCRs were set up in 25 μl reaction volumes containing 14.3 μl AccuGENE® water, 0.2 μl AmpliTaq Gold®, 2.5 μl 10× PCR buffer, 2.5 μl MgCl_2_ (25 mM), 2.5 μl dNTPs (2 mM each), 2 μl DNA template and 0.5 μl of each of the respective primers (10 μM each). An initial denaturation step of 5 min at 95°C was followed by 40 cycles (95°C for 40 s; 55°C (*COI*) or 64°C (*wg*) for 40 s; 72°C for 40 s) and a final extension of 15 min at 72°C using an Eppendorf Mastercycler® proS. Purification and sequencing of PCR products was performed by commercial facilities (Macrogen USA or Eton Bioscience). All PCR products were sequenced in both directions. Sequences are deposited under accession numbers KM576918–KM577157 (*COI*) and KM591602–KM591681 (*wg*) in GenBank (Supplementary Tab. S1).

### Data management and analysis

The laboratory information management system (LIMS) implemented in the software Geneious® (version 7.1.7) with the biocode plugin (version 2.8.0) was used to track all workflows including collection data, extractions, PCRs, and cycle sequencing^84^. Sequences were aligned and trimmed in Geneious®. Neighbor-joining trees based on Tamura-Nei distances with bootstrap support (1,000 replicates) were created using Geneious® Tree Builder and a Randomized Axelerated Maximum Likelihood^85^ tree (RAxML; version 8.1.X) was created using the GTR + gamma model. MEGA 6^86^ was used to assess number of variable, parsimony-informative sites and singletons and to calculate p-distances with gaps deleted in pairwise comparisons. Tree-Parser-aided Klee diagrams were created as described in Stoeckle and Coffran (2013)^87^. The level of genetic differentiation for the nuclear gene *wg* among different *COI* haplogroups was calculated as pairwise F_ST_ in the software FSTAT 2.9.3.2^88^ following Weir and Cockerham (1984)^89^. Statistical significance assuming Hardy-Weinberg equilibrium was assessed using randomization tests with 10,000 iterations.

Five *wg* alleles segregating in the population were directly observed in homozygous individuals. Three additional alleles were inferred from heterozygous individuals by subtracting one of the five alleles observed in the homozygous state. In the vast majority of heterozygous individuals, the chromatogram could only be explained by a single combination of two of these eight alleles. Only in two heterozygous individuals was there ambiguity over the respective allele combination, and therefore we omitted those individuals from the F_ST_ analysis.

## Supplementary Material

Supplementary Information Supplementary Information

## References

[b1] Simberloff D. Invasive Species: What everyone needs to know. (Oxford University Press, New York, 2013).

[b2] Hulme P. E. Trade, transport and trouble: managing invasive species pathways in an era of globalization. J. Appl. Ecol. 46, 10–18, 10.1111/j.1365-2664.2008.01600.x (2009).

[b3] Armstrong K. F. & Ball S. L. DNA barcodes for biosecurity: invasive species identification. Philos. Trans. R. Soc. Lond. B. Biol. Sci. 360, 1813–1823, 10.1098/rstb.2005.1713 (2005). 16214740 PMC1609225

[b4] Darling J. A. & Blum M. J. DNA-based methods for monitoring invasive species: a review and prospectus. Biol. Invasions 9, 751–765, 10.1007/s10530-006-9079-4 (2007).

[b5] Rubinoff D. Utility of mitochondrial DNA barcodes in species conservation. Conserv. Biol. 20, 1026–1033, 10.1111/j.1523-1739.2006.00372.x (2006). 16922219

[b6] Krishnamurthy P. K. & Francis R. A. A critical review on the utility of DNA barcoding in biodiversity conservation. Biodivers. Conserv. 21, 1901–1919, 10.1007/s10531-012-0306-2 (2012).

[b7] Meier R., Zhang G. & Ali F. The use of mean instead of smallest interspecific distances exaggerates the size of the “Barcoding Gap” and leads to misidentification. Syst. Biol. 57, 809–813, 10.1080/10635150802406343 (2008). 18853366

[b8] Wiemers M. & Fiedler K. Does the DNA barcoding gap exist? - a case study in blue butterflies (Lepidoptera: Lycaenidae). Front. Zool. 4, 8–8, 10.1186/1742-9994-4-8 (2007). 17343734 PMC1838910

[b9] Waugh J. DNA barcoding in animal species: progress, potential and pitfalls. Bioessays 29, 188–197, 10.1002/bies.20529 (2007). 17226815

[b10] Hebert P. D. N., Ratnasingham S. & deWaard J. R. Barcoding animal life: *cytochrome c oxidase* subunit 1 divergences among closely related species. Proc. Roy. Soc. B 270, S96–S99, 10.1098/rsbl.2003.0025 (2003). PMC1698023 12952648

[b11] Hajibabaei M., Singer G. A. C., Hebert P. D. N. & Hickey D. A. DNA barcoding: how it complements taxonomy, molecular phylogenetics and population genetics. Trends Genet. 23, 167–172 (2007). 17316886 10.1016/j.tig.2007.02.001

[b12] Goldstein P. Z. & DeSalle R. Integrating DNA barcode data and taxonomic practice: Determination, discovery, and description. Bioessays 33, 135–147, 10.1002/bies.201000036 (2011). 21184470

[b13] Jinbo U., Kato T. & Ito M. Current progress in DNA barcoding and future implications for entomology. Entomol. Sci. 14, 107–124, 10.1111/j.1479-8298.2011.00449.x (2011).

[b14] Ratnasingham S. & Hebert P. D. N. BOLD: The Barcode of Life Data System (www.barcodinglife.org) Mol. Ecol. Notes 7, 355–364, 10.1111/j.1471-8286.2006.01678.x (2007). 18784790 PMC1890991

[b15] Ratnasingham S. & Hebert P. D. N. BOLD's role in barcode data management and analysis: a response. Mol. Ecol. Res. 11, 941–942, 10.1111/j.1755-0998.2011.03067.x (2011).

[b16] Hebert P. D. N., Stoeckle M. Y., Zemlak T. S. & Francis C. M. Identification of birds through DNA barcodes. PLoS Biol. 2, 1657–1663, 10.1371/journal.pbio.0020312 (2004). PMC518999 15455034

[b17] Hebert P. D. N., Penton E. H., Burns J. M., Janzen D. H. & Hallwachs W. Ten species in one: DNA barcoding reveals cryptic species in the neotropical skipper butterfly *Astraptes fulgerator*. PNAS 101, 14812–14817, 10.1073/pnas.0406166101 (2004). 15465915 PMC522015

[b18] Hajibabaei M., Janzen D. H., Burns J. M., Hallwachs W. & Hebert P. D. N. DNA barcodes distinguish species of tropical Lepidoptera. PNAS 103, 968–971, 10.1073/pnas.0510466103 (2006). 16418261 PMC1327734

[b19] Smith M. A. *et al.* Extreme diversity of tropical parasitoid wasps exposed by iterative integration of natural history, DNA barcoding, morphology, and collections. PNAS 105, 12359–12364, 10.1073/pnas.0805319105 (2008). 18716001 PMC2518452

[b20] Ward R. D., Zemlak T. S., Innes B. H., Last P. R. & Hebert P. D. N. DNA barcoding Australia's fish species. Philos. Trans. R. Soc. Lond. B. Biol. Sci. 360, 1847–1857, 10.1098/rstb.2005.1716 (2005). 16214743 PMC1609232

[b21] Resch M. C. *et al.* Where taxonomy based on subtle morphological differences is perfectly mirrored by huge genetic distances: DNA Barcoding in protura (Hexapoda). PLoS One 9, 10.1371/journal.pone.0090653 (2014). PMC3946556 24609003

[b22] Scheffer S. J., Lewis M. L. & Joshi R. C. DNA barcoding applied to invasive leafminers (Diptera: Agromyzidae) in the Philippines. Ann. Entomol. Soc. Am. 99, 204–210, 10.1603/0013-8746(2006)099[0204:dbatil]2.0.co;2 (2006).

[b23] Smith P. J., Webber W. R., McVeagh S. M., Inglis G. J. & Gust N. DNA and morphological identification of an invasive swimming crab, *Charybdis japonica*, in New Zealand waters. New Zeal. J. Mar. Fresh. 37, 753–762 (2003).

[b24] Ficetola G. F., Bonin A. & Miaud C. Population genetics reveals origin and number of founders in a biological invasion. Mol. Ecol. 17, 773–782 (2008). 18194168 10.1111/j.1365-294X.2007.03622.x

[b25] Saunders G. W. Routine DNA barcoding of Canadian Gracilariales (Rhodophyta) reveals the invasive species *Gracilaria vermiculophylla* in British Columbia. Mol. Ecol. Res. 9, 140–150, 10.1111/j.1755-0998.2009.02639.x (2009). 21564973

[b26] Facon B. *et al.* A molecular phylogeography approach to biological invasions of the New World by parthenogenetic Thiarid snails. Mol. Ecol. 12, 3027–3039, 10.1046/j.1365-294X.2003.01972.x (2003). 14629383

[b27] Marescaux J. & Van Doninck K. Using DNA barcoding to differentiate invasive *Dreissena* species (Mollusca, Bivalvia). Zookeys, 235–244, 10.3897/zookeys.365.5905 (2013). 24453560 PMC3890680

[b28] Simonsen T. J., Brown R. L. & Sperling F. A. H. Tracing an invasion: Phylogeography of *Cactoblastis cactorum* (Lepidoptera: Pyralidae) in the United States based on mitochondrial DNA. Ann. Entomol. Soc. Am. 101, 899–905, 10.1603/0013-8746(2008)101[899:taipoc]2.0.co;2 (2008).

[b29] Nadel R. L. *et al.* DNA bar-coding reveals source and patterns of *Thaumastocoris peregrinus* invasions in South Africa and South America. Biol. Invasions 12, 1067–1077, 10.1007/s10530-009-9524-2 (2010).

[b30] Rubinoff D., Holland B. S., Shibata A., Messing R. H. & Wright M. G. Rapid invasion despite lack of genetic variation in the erythrina gall wasp (*Quadrastichus erythrinae* Kim). Pac. Sci. 64, 23–31, 10.2984/64.1.023 (2010).

[b31] Roman J. & Darling J. A. Paradox lost: genetic diversity and the success of aquatic invasions. Tr. Ecol. Evol. 22, 454–464, 10.1016/j.tree.2007.07.002 (2007). 17673331

[b32] Dlugosch K. M. & Parker I. M. Founding events in species invasions: genetic variation, adaptive evolution, and the role of multiple introductions. Mol. Ecol. 17, 431–449, 10.1111/j.1365-294X.2007.03538.x (2008). 17908213

[b33] Bell W., Roth L. & Nalepa C. Cockroaches: Ecology, behavior, and natural history. (The Johns Hopkins University Press, Baltimore, 2007).

[b34] Guthrie D. & Tindall A. The biology of the cockroach. (St. Martin's Press, New York, 1968).

[b35] Bell W. J. & Adiyodi K. G. The American cockroach. (Chapman and Hall Ltd, New York 1981).

[b36] Rehn J. A. G. Man's uninvited fellow travellers – the cockroach. Sci. Mon. 61, 265–276 (1945).

[b37] Bonnefoy X., Kampen H. & Sweeney K. Public Health Significance Of Urban Pests. (World Health Organization, Copenhagen, 2008).

[b38] Appel A. G. & Smith L. M. Biology and management of the smokybrown cockroach. Ann. Rev. Entomol. 47, 33–55, 10.1146/annurev.ento.47.091201.145106 (2002). 11729068

[b39] Iwuala M. O. E., Osisiogu I. U. W. & Agbakwuru E. O. P. Dennetia oil, a potential new insecticide: tests with adults and nymphs of *Periplaneta americana* and *Zonocerus variegatus*. J. Econ. Entomol. 74, 249–52 (1981).

[b40] Schal C. & Hamilton R. L. Integrated supression of synanthropic cockroaches. Ann. Rev. Entomol. 35, 521–551 (1990). 2405773 10.1146/annurev.en.35.010190.002513

[b41] Shahraki G., Parhizkar S. & Nejad A. Cockroach infestation and factors affecting the estimation of cockroach population in urban communities. Internat. J. Zool. 10.1155/2013/649089 (2013).

[b42] Gade G. & Goldsworthy G. J. Insect peptide hormones: a selective review of their physiology and potential application for pest control. Pest Manag. Sci. 59, 1063–1075, 10.1002/ps.755 (2003). 14561063

[b43] Lihoreau M., Costa J. T. & Rivault C. The social biology of domiciliary cockroaches: colony structure, kin recognition and collective decisions. Insect. Soc. 59, 445–452, 10.1007/s00040-012-0234-x (2012).

[b44] Whyatt R. M. *et al.* Residential pesticide use during pregnancy among a cohort of urban minority women. Environ. Health Perspect. 110, 507–514 (2002). 12003754 10.1289/ehp.02110507 PMC1240839

[b45] Evangelista D., Buss L. & Ware J. Using DNA barcodes to confirm the presence of a new invasive cockroach pest in New York City. J. Eco. Entomol. 106, 2275–2279 (2013). 10.1603/ec13402 24498724

[b46] Brenner B. L. *et al.* Integrated pest management in an urban community: A successful partnership for prevention. Environ. Health Perspect. 111, 1649–1653, 10.1289/ehp.6069 (2003). 14527845 PMC1241688

[b47] Marshall D. C., Hill K. B. R., Cooley J. R. & Simon C. Hybridization, mitochondrial DNA phylogeography, and prediction of the early stages of reproductive isolation: Lessons from New Zealand cicadas (genus Kikihia). Syst. Biol. 60, 482–502, 10.1093/sysbio/syr017 (2011). 21471306

[b48] Zhang A.-B. & Sota T. Nuclear gene sequences resolve species phylogeny and mitochondrial introgression in *Leptocarabus* beetles showing trans-species polymorphisms. Mol. Phyl. Evol. 45, 534–546, 10.1016/j.ympev.2007.07.003 (2007). 17693098

[b49] McGuire J. A. *et al.* Mitochondrial introgression and incomplete lineage sorting through space and time: Phylogenetics of crotaphytid lizards. Evolution 61, 2879–2897, 10.1111/j.1558-5646.2007.00239.x (2007). 17941840

[b50] Whitworth T. L., Dawson R. D., Magalon H. & Baudry E. DNA barcoding cannot reliably identify species of the blowfly genus *Protocalliphora* (Diptera: Calliphoridae). Proc. Roy. Soc. B 274, 1731–1739, 10.1098/rspb.2007.0062 (2007). PMC2493573 17472911

[b51] Vences M., Thomas M., Bonett R. M. & Vieites D. R. Deciphering amphibian diversity through DNA barcoding: chances and challenges. Phil. Trans. Roy. Soc. B 360, 1859–1868, 10.1098/rstb.2005.1717 (2005). 16221604 PMC1609216

[b52] Moritz C., Dowling T. E. & Brown W. M. Evolution of animal mitochondrial-DNA - Relevance for population biology and systematics. Ann. Rev. Ecol. Syst. 18, 269–292, 10.1146/annurev.es.18.110187.001413 (1987).

[b53] Ballard J. W. O. & Whitlock M. C. The incomplete natural history of mitochondria. Mol. Ecol. 13, 729–744, 10.1046/j.1365-294X.2003.02063.x (2004). 15012752

[b54] Ritter S. *et al.* *Wolbachia* infections mimic cryptic speciation in two parasitic butterfly species, *Phengaris teleius* and *P. nausithous* (Lepidoptera: Lycaenidae). PloS One 8, 10.1371/journal.pone.0078107 (2013). PMC3819333 24223136

[b55] Hurst G. D. D. & Jiggins F. M. Problems with mitochondrial DNA as a marker in population, phylogeographic and phylogenetic studies: the effects of inherited symbionts. Proc. Roy. Soc. B 272, 1525–1534, 10.1098/rspb.2005.3056 (2005). PMC1559843 16048766

[b56] Munoz A. G., Baxter S. W., Linares M. & Jiggins C. D. Deep mitochondrial divergence within a Heliconius butterfly species is not explained by cryptic speciation or endosymbiotic bacteria. Bmc Evol. Biol. 11 10.1186/1471-2148-11-358 (2011). PMC3287262 22151691

[b57] Zamudio K. R. & Savage W. K. Historical isolation, range expansion, and secondary contact of two highly divergent mitochondrial lineages in spotted salamanders (*Ambystoma maculatum*). Evolution 57, 1631–1652 (2003). 12940367 10.1554/02-342

[b58] Candek K. & Kuntner M. DNA barcoding gap: reliable species identification over morphological and geographical scales. Mol. Ecol. Res. 10.1111/1755-0998.12304 (2014). 25042335

[b59] Pereira L. H. G., Hanner R., Foresti F. & Oliveira C. Can DNA barcoding accurately discriminate megadiverse Neotropical freshwater fish fauna? Bmc Genet. 14, 10.1186/1471-2156-14-20 (2013). PMC3608943 23497346

[b60] Burns J. M., Janzen D. H., Hajibabaei M., Hallwachs W. & Hebert P. D. N. DNA barcodes of closely related (but morphologically and ecologically distinct) species of skipper butterflies (Hesperiidae) can differ by only one to three nucleotides. J. Lepidopt. Soc. 61, 138–153 (2007).

[b61] Elias M. *et al.* Limited performance of DNA barcoding in a diverse community of tropical butterflies. Proc. Roy. Soc. B. 274, 2881–2889, 10.1098/rspb.2007.1035 (2007). PMC3227132 17785265

[b62] Serbus L. R., Casper-Lindley C., Landmann F. & Sullivan W. The genetics and cell biology of *Wolbachia*-host interactions. Ann. Rev. Genet. 42, 683–707, 10.1146/annurev.genet.41.110306.130354 (2008). 18713031

[b63] Russell J. A. The ants (Hymenoptera: Formicidae) are unique and enigmatic hosts of prevalent *Wolbachia* (Alphaproteobacteria) symbionts. Myrmecol. News 16, 7–23 (2012).

[b64] Vaishampayan P. A. *et al.* Molecular evidence and phylogenetic affiliations of *Wolbachia* in cockroaches. *Mol*. Phylogenet. Evol. 44, 1346–1351, 10.1016/j.ympev.2007.01.003 (2007). 17350292

[b65] Jiggins F. M. Male-killing *Wolbachia* and mitochondrial DNA: Selective sweeps, hybrid introgression and parasite population dynamics. Genetics 164, 5–12 (2003). 12750316 10.1093/genetics/164.1.5 PMC1462540

[b66] Xiao J.-H. *et al.* *Wolbachia* infection and dramatic intraspecific mitochondrial DNA divergence in a fig wasp. Evolution 66, 1907–1916, 10.1111/j.1558-5646.2011.01561.x (2012). 22671555

[b67] Papadopoulou A., Anastasiou I. & Vogler A. P. Revisiting the insect mitochondrial molecular clock: The mid-Aegean trench calibration. Mol. Biol. Evol. 27, 1659–1672, 10.1093/molbev/msq051 (2010). 20167609

[b68] Henderson I. & Robertson P. Control and eradication of the North American ruddy duck in Europe. Manag. Vert. Invas. Sp. paper 16, http://digitalcommons.unl.edu/nwrcinvasive/16 (2007).

[b69] Frankham R. Invasion biology - Resolving the genetic paradox in invasive species. Heredity 94, 385–385, 10.1038/sj.hdy.6800634 (2005). 15602569

[b70] Simberloff D. The role of propagule pressure in biological invasions. Ann. Rev. Ecol. Evol. Syst. 40, 81–102, 10.1146/annurev.ecolsys.110308.120304 (2009).

[b71] Lavergne S. & Molofsky J. Increased genetic variation and evolutionary potential drive the success of an invasive grass. PNAS 104, 3883–3888, 10.1073/pnas.0607324104 (2007). 17360447 PMC1805698

[b72] Verhoeven K. J. F., Macel M., Wolfe L. M. & Biere A. Population admixture, biological invasions and the balance between local adaptation and inbreeding depression. Proc. Roy. Soc. B 278, 2–8, 10.1098/rspb.2010.1272 (2011). PMC2992731 20685700

[b73] Facon B. *et al.* Can things get worse when an invasive species hybridizes? The harlequin ladybird *Harmonia axyridis* in France as a case study. Evol. Appl. 4, 71–88, 10.1111/j.1752-4571.2010.00134.x (2011). 25567954 PMC3352518

[b74] Turgeon J. *et al.* Experimental evidence for the phenotypic impact of admixture between wild and biocontrol Asian ladybird (*Harmonia axyridis*) involved in the European invasion. J. Evol. Biol. 24, 1044–1052, 10.1111/j.1420-9101.2011.02234.x (2011). 21342302

[b75] Keller L. F. & Waller D. M. Inbreeding effects in wild populations. Tr. Ecol Evol. 17, 230–241, 10.1016/s0169-5347(02)02489-8 (2002).

[b76] Crnokrak P. & Roff D. A. Inbreeding depression in the wild. Heredity 83, 260–270, 10.1038/sj.hdy.6885530 (1999). 10504423

[b77] Charlesworth D. & Charlesworth B. Inbreeding depression and its evolutionary consequences. Ann. Rev. Ecol. Syst. 18, 237–268, 10.1146/annurev.ecolsys.18.1.237 (1987).

[b78] Frankham R. & Ralls K. Conservation biology - Inbreeding leads to extinction. Nature 392, 441–442, 10.1038/33022 (1998).

[b79] Saccheri I. *et al.* Inbreeding and extinction in a butterfly metapopulation. Nature 392, 491–494, 10.1038/33136 (1998).

[b80] Cooling M., Hartley S., Sim D. A. & Lester P. J. The widespread collapse of an invasive species: Argentine ants (*Linepithema humile*) in New Zealand. Biol. Lett. 8(3), 430–433, 10.1098/rsbl.2011.1014 (2012). 22130172 PMC3367738

[b81] Helfer J. R. How to Know the Grasshoppers, Crickets, Cockroaches and Their Allies. [35–59] (Dover Publications, New York, 1987).

[b82] Folmer O., Black M., Hoeh W., Lutz R. & Vrijenhoek R. DNA primers for amplification of mitochondrial cytochrome c oxidase subunit I from diverse metazoan invertebrates. Mol. Mar. Biol. Biotech. 3, 294–299 (1994). 7881515

[b83] Wild A. L. & Maddison D. R. Evaluating nuclear protein-coding genes for phylogenetic utility in beetles. Mol. Phylogen. Evol. 48, 877–891, 10.1016/j.ympev.2008.05.023 (2008). 18644735

[b84] Parker M., Stones-Havas S., Starger C. & Meyer C. Laboratory information management systems for DNA barcoding. Meth. Mol. Biol. 858, 269–310, 10.1007/978-1-61779-591-6_13 (2012). 22684961

[b85] Stamatakis A. RAxML version 8: a tool for phylogenetic analysis and post-analysis of large phylogenies. Bioinformatics 30, 1312–1313, 10.1093/bioinformatics/btu033 (2014). 24451623 PMC3998144

[b86] Tamura K., Stecher G., Peterson D., Filipski A. & Kumar S. MEGA6: Molecular Evolutionary Genetics Analysis Version 6.0. Mol. Biol. Evol. 30, 2725–2729, 10.1093/molbev/mst197 (2013). 24132122 PMC3840312

[b87] Stoeckle M. Y. & Coffran C. TreeParser-aided Klee diagrams display taxonomic clusters in DNA barcode and nuclear gene datasets. Sci. Rep. 3, 10.1038/srep02635 (2013). PMC3769653 24022383

[b88] Goudet J. FSTAT, a program to estimate and test gene diversities and fixation indices (version 2.9.3.2) Available at: http://www2.unil.ch/popgen/softwares/fstat.htm(2001). Date of access: 05/05/2014.

[b89] Weir B. S. & Cockerham C. C. Estimating F-statistics for the analysis of population structure. Evolution 38, 1358–1370, 10.2307/2408641 (1984). 28563791

