# Supplementary material for: Interbreeding among deeply divergent mitochondrial lineages in the American cockroach (Periplaneta americana)
Source: Sci Rep. 2015 Feb 6;5:8297. doi: 10.1038/srep08297 (PMC4650827; doi:10.1038/srep08297)
Supplement: Supplementary Information [file srep08297-s1.pdf]

TITLE: Interbreeding among deeply divergent mitochondrial lineages in the American cockroach (*Periplaneta americana*)

AUTHORS: Christoph von Beeren, Mark Y. Stoeckle, Joyce Xia, Griffin Burke, and Daniel J.C. Kronauer

Table S1. Cockroach specimens catalogued in this study (N=284).

Table S2. Previous GenBank records of *P. americana COI* barcodes included in the analyses (N=24) and of other cockroaches used as reference sequences for species identifications.

Figure S1. Randomized Axelerated Maximum Likelihood (RAxML) tree for mitochondrial *COI* sequences of *Periplaneta* species.

Table S1. Cockroach specimens catalogued in this study (N=284). Abbreviations: *Gromphadorhina potentosa* (Gpo), *Periplaneta americana* (Pam), *P. fuliginosa* (Pfu), *Shellfordella lateralis* (Sla), *Supella longipalpa* (Slo), Coleoptera (Col) identified via BOLD search as *Cyclocephala borealis* (99.85% sequence similarity); *cytochrome oxidase I* (*COI*), *wingless* (*wg*), American Museum of Natural History (AMNH), n/a = not applicable.

Specimen ID	Amplicon full-length (F), short (S), or none (N)	Species ID	Haplotype (Pam only)	City	Country States	Country	U.S. zip code	Collection date (month/day/ year)	Unique voucher ID (AMNH)	GenBank accession number (*COI*)	GenBank accession number (*wg*)
**JX001**	F	Pam	C1	Boston	MA	USA	02121	06/07/13	n/a	KM577123	KM591645
**JX002**	F	Pam	A1	Boston	MA	USA	02121	06/07/13	n/a	KM576927	KM591621
**JX003**	F	Pfu	n/a	Cayce	SC	USA	29033	06/11/13	n/a	KM576992	n/a
**JX004**	F	Pam	A1	New York	NY	USA	10065	06/19/13	n/a	KM577134	n/a
**JX005**	N	n/a	n/a	Kuranda	QLD	Australia	n/a	05/31/13	n/a	n/a	n/a
**JX006**	F	Pam	B1	New York	NY	USA	10065	June 2013	n/a	KM577106	KM591652
**JX007**	F	Pam	C1	New York	NY	USA	11222	06/24/13	n/a	KM577139	KM591640
**JX008**	F	Pam	C1	New York	NY	USA	11222	06/24/13	AMNH_IZC 00114343	KM576921	KM591663
**JX009**	S	Bge	n/a	New York	NY	USA	10462	12/19/12	n/a	KM577129	n/a
**JX010**	F	Pam	C1	New Orleans	LA	USA	70112	11/03/12	n/a	KM577117	KM591613
**JX011**	F	Pam	A1	New York	NY	USA	10065	06/12/13	n/a	KM576950	-
**JX012**	F	Pam	A1	New York	NY	USA	10065	06/12/13	n/a	KM577029	-
**JX013**	F	Pam	A1	New York	NY	USA	10065	06/12/13	n/a	KM576942	KM591641
**JX014**	F	Pam	A1	New York	NY	USA	10065	06/12/13	n/a	KM576978	KM591653
**JX015**	F	Pam	A1	New York	NY	USA	10065	06/12/13	n/a	KM576979	KM591604
**JX016**	F	Pam	A1	New York	NY	USA	10065	06/12/13	n/a	KM577130	n/a
**JX017**	F	Pam	A1	New York	NY	USA	10065	06/12/13	n/a	KM577113	KM591666
**JX018**	F	Pam	A1	New York	NY	USA	10065	06/12/13	n/a	KM577046	n/a
**JX019**	F	Pam	B1	New York	NY	USA	10065	06/12/13	n/a	KM576998	n/a
**JX020**	F	Pam	A1	New York	NY	USA	10065	06/12/13	n/a	KM577118	KM591678
**JX021**	F	Pam	A1	New York	NY	USA	10065	06/12/13	n/a	KM576952	n/a
**JX022**	F	Pam	A1	New York	NY	USA	10065	06/12/13	n/a	KM576974	KM591647
**JX023**	F	Pam	A1	New York	NY	USA	10065	06/12/13	n/a	KM576964	KM591615
**JX024**	F	Pam	A1	New York	NY	USA	10065	06/12/13	n/a	KM576975	n/a
**JX025**	F	Pam	A1	New York	NY	USA	10065	06/12/13	n/a	KM577091	KM591660
**JX026**	F	Pam	A3	New York	NY	USA	10065	06/12/13	AMNH_IZC 00114341	KM576999	n/a
**JX027**	F	Pam	A1	New York	NY	USA	10065	06/12/13	n/a	KM576947	KM591674
**JX028**	F	Pam	A1	New York	NY	USA	10065	06/12/13	n/a	KM577025	KM591669
**JX029**	F	Pam	A1	New York	NY	USA	10065	06/12/13	n/a	KM577048	n/a
**JX030**	F	Pam	A3	New York	NY	USA	10065	06/12/13	n/a	KM577115	n/a
**JX031**	F	Pam	B1	New York	NY	USA	10065	06/12/13	n/a	KM577136	n/a
**JX032**	F	Pam	C1	New York	NY	USA	10065	06/12/13	n/a	KM576925	KM591607
**JX033**	F	Pam	A1	New York	NY	USA	10065	06/05/13	n/a	KM576922	KM591630
**JX034**	F	Pam	A1	New York	NY	USA	10028	06/05/13	n/a	KM577063	n/a
**JX035**	F	Pam	C1	New York	NY	USA	10065	06/25/13	AMNH_IZC 00114342	KM577011	KM591670
**JX036**	F	Pam	B1	New York	NY	USA	10065	06/04/13	n/a	KM577098	KM591677
**JX037**	F	Pam	B1	New York	NY	USA	10065	06/04/13	n/a	KM577145	n/a
**JX038**	F	Pam	B1	New York	NY	USA	10065	05/14/13	n/a	KM577054	KM591661
**JX039**	F	Pam	B1	New York	NY	USA	10065	06/04/13	n/a	KM577072	n/a
**JX040**	F	Pam	A3	New York	NY	USA	10024	04/22/13	n/a	KM577086	KM591654
**JX041**	S	Bge	n/a	New York	NY	USA	10025	03/19/13	n/a	n/a	n/a
**JX042**	F	Pam	A1	New York	NY	USA	10065	06/11/13	n/a	KM576944	n/a
**JX043**	S	Bge	n/a	New York	NY	USA	11215	06/06/13	n/a	n/a	n/a
**JX044**	F	Pam	B1	New York	NY	USA	10065	06/11/13	n/a	KM576977	n/a
**JX045**	F	Pam	B1	New York	NY	USA	10065	06/11/13	n/a	KM577030	n/a
**JX046**	F	Pam	B1	New York	NY	USA	10065	06/11/13	n/a	KM576941	n/a

**JX047**	N	n/a	n/a	New York	NY	USA	10065	06/11/13	n/a	n/a	n/a
**JX048**	F	Pfu	n/a	Evans	GA	USA	30907	06/07/13	n/a	KM577119	n/a
**JX049**	F	Pam	B1	New York	NY	USA	10065	06/10/13	n/a	KM576918	n/a
**JX050**	N	n/a	n/a	New York	NY	USA	10065	06/10/13	n/a	n/a	n/a
**JX051**	N	n/a	n/a	Washington DC	DC	USA	20036	06/08/13	n/a	n/a	n/a
**JX052**	F	Pam	B1	New York	NY	USA	10065	06/28/13	n/a	KM576946	n/a
**JX053**	F	Pam	C1	New York	NY	USA	11216	06/28/13	n/a	KM576924	KM591626
**JX054**	F	Pam	A2	New York	NY	USA	10471	06/25/13	AMNH_IZC 00114350	KM577122	KM591658
**JX055**	F	Pam	C1	Harvey	LA	USA	70058	06/17/13	n/a	KM577081	KM591675
**JX056**	F	Pam	A5	Harvey	LA	USA	70058	06/21/13	n/a	KM577024	KM591676
**JX057**	F	Pam	A1	New York	NY	USA	10024	07/02/13	n/a	KM577028	n/a
**JX058**	N	n/a	n/a	New York	NY	USA	10065	06/26/13	n/a	n/a	n/a
**JX059**	F	Pam	C1	New York	NY	USA	10024	07/03/13	n/a	KM577104	KM591639
**JX060**	F	Pam	A1	New York	NY	USA	10065	07/08/13	n/a	KM576965	KM591616
**JX061**	F	Pam	A3	New York	NY	USA	10065	07/11/13	n/a	KM577084	n/a
**JX062**	F	Pam	A3	New York	NY	USA	10065	07/11/13	n/a	KM577088	KM591667
**JX063**	F	Pam	B1	New York	NY	USA	10065	07/11/13	n/a	KM576919	n/a
**JX064**	F	Pam	B1	New York	NY	USA	10065	07/12/13	n/a	KM576954	n/a
**JX065**	F	Pam	C1	New York	NY	USA	10024	07/09/13	n/a	KM577000	KM591632
**JX066**	F	Pam	B1	New York	NY	USA	10021	07/11/13	n/a	KM577078	KM591611
**JX067**	F	Pam	C1	New York	NY	USA	10024	07/15/13	n/a	KM576936	n/a
**JX068**	S	Bge	n/a	New York	NY	USA	10044	07/16/13	n/a	n/a	n/a
**JX069**	S	Bge	n/a	New York	NY	USA	10044	07/16/13	n/a	n/a	n/a
**JX070**	S	Bge	n/a	New York	NY	USA	10044	07/16/13	n/a	n/a	n/a
**JX071**	F	Pam	A1	Burlington	NC	USA	27215	07/15/13	AMNH_IZC 00114351	KM577014	n/a
**JX072**	F	Pam	A3	Burlington	NC	USA	27215	07/15/13	n/a	KM576990	n/a
**JX073**	S	Bge	n/a	New York	NY	USA	10024	07/15/13	n/a	n/a	n/a
**JX074**	S	Bge	n/a	New York	NY	USA	10024	07/15/13	n/a	n/a	n/a
**JX075**	F	Pam	C1	New York	NY	USA	10024	07/16/13	n/a	KM577035	KM591671
**JX076**	F	Pam	C1	New York	NY	USA	10044	07/15/13	n/a	KM577100	KM591638
**JX077**	F	Pam	A1	Washington DC	DC	USA	20013	07/08/13	n/a	KM577154	KM591608
**JX078**	F	Pam	A1	New York	NY	USA	10065	07/22/13	n/a	KM577053	n/a
**JX079**	F	Pam	A1	New York	NY	USA	10065	07/22/13	n/a	KM577127	n/a
**JX080**	F	Pam	A3	New York	NY	USA	10024	07/22/13	n/a	KM577107	KM591665
**JX081**	F	Pam	C1	La Coruña	n/a	Spain	15006	07/08/13	n/a	KM576962	KM591656
**JX082**	F	Pam	C1	La Coruña	n/a	Spain	15006	07/08/13	n/a	KM576997	KM591643
**JX083**	F	Pam	A1	New York	NY	USA	10065	07/17/13	n/a	KM577094	n/a
**JX084**	F	Pam	B2	New York	NY	USA	10044	07/18/13	n/a	KM577015	KM591664
**JX085**	F	Pam	B2	New York	NY	USA	10044	07/18/13	n/a	KM577146	KM591623
**JX086**	F	Col	n/a	New York	NY	USA	11354	07/22/13	n/a	KM577076	n/a
**JX087**	F	Pam	A1	New York	NY	USA	10065	07/26/13	n/a	KM576973	n/a
**JX088**	F	Pam	A1	New York	NY	USA	10065	07/26/13	n/a	KM577070	n/a
**JX089**	F	Pam	A1	New York	NY	USA	10065	07/19/13	AMNH_IZC 00114349	KM576980	n/a
**JX090**	F	Pam	C1	New York	NY	USA	10044	07/22/13	n/a	KM576961	KM591631
**JX091**	F	Pam	B2	New York	NY	USA	10044	07/19/13	n/a	KM577003	KM591681
**JX092**	F	Pam	B2	New York	NY	USA	10044	07/22/13	n/a	KM577128	KM591627
**JX093**	F	Pam	B2	New York	NY	USA	10044	07/22/13	n/a	KM576968	KM591648
**JX094**	F	Pam	B2	New York	NY	USA	10044	07/22/13	n/a	KM577038	KM591610
**JX095**	F	Pam	B2	New York	NY	USA	10044	07/22/13	AMNH_IZC 00114338	KM576931	KM591606

**JX096**	F	Pam	B2	New York	NY	USA	10044	07/22/13	n/a	KM577050	KM591659
**JX097**	F	Pam	C1	New York	NY	USA	10044	07/23/13	AMNH_IZC 00114336	KM576929	KM591644
**JX098**	F	Pam	A2	New York	NY	USA	10044	07/23/13	n/a	KM576932	KM591679
**JX099**	F	Pam	B2	New York	NY	USA	10044	07/23/13	n/a	KM577116	KM591603
**JX100**	F	Pam	B2	New York	NY	USA	10044	07/23/13	n/a	KM577143	KM591625
**JX101**	F	Pam	B2	New York	NY	USA	10011	07/01/13	n/a	KM577137	KM591655
**JX102**	F	Pam	B2	New York	NY	USA	10011	07/01/13	n/a	KM576993	KM591629
**JX103**	F	Pam	B2	New York	NY	USA	10024	07/25/13	n/a	KM577087	KM591602
**JX104**	F	Pam	A1	New York	NY	USA	10128	07/26/13	n/a	KM577033	KM591612
**JX105**	N	n/a	n/a	New York	NY	USA	10065	07/26/13	n/a	n/a	n/a
**JX106**	F	Pam	B2	New York	NY	USA	10044	07/26/13	n/a	KM577066	KM591668
**JX107**	F	Pam	B2	New York	NY	USA	10044	07/26/13	n/a	KM577090	KM591637
**JX108**	N	n/a	n/a	New York	NY	USA	10044	07/26/13	n/a	n/a	n/a
**JX109**	F	Pam	B2	New York	NY	USA	10044	07/26/13	n/a	KM577058	KM591636
**JX110**	F	Pam	B2	New York	NY	USA	10044	07/26/13	n/a	KM577004	KM591605
**JX111**	F	Pam	B2	New York	NY	USA	10044	07/26/13	n/a	KM577109	KM591673
**JX112**	F	Pam	B2	New York	NY	USA	10044	07/26/13	AMNH_IZC 00114339	KM576986	KM591662
**JX113**	F	Pam	B2	New York	NY	USA	10044	07/26/13	n/a	KM577023	KM591634
**JX114**	F	Pam	B2	New York	NY	USA	10044	07/26/13	n/a	KM577099	KM591646
**JX115**	F	Pam	B2	New York	NY	USA	10044	07/26/13	n/a	KM577010	n/a
**JX116**	F	Pam	B2	New York	NY	USA	10044	07/26/13	n/a	KM577111	n/a
**JX117**	F	Pam	B2	New York	NY	USA	10044	07/26/13	AMNH_IZC 00114340	KM576969	n/a
**JX118**	F	Pam	B2	New York	NY	USA	10044	07/26/13	n/a	KM577085	n/a
**JX119**	F	Pam	B2	New York	NY	USA	10044	07/26/13	AMNH_IZC 00114337	KM577120	n/a
**JX120**	F	Pam	B2	New York	NY	USA	10044	07/26/13	n/a	KM576951	n/a
**JX121**	F	Pam	B2	New York	NY	USA	10044	07/26/13	n/a	KM577026	n/a
**JX122**	N	n/a	n/a	New York	NY	USA	10044	07/26/13	n/a	n/a	n/a
**JX123**	F	Pam	B2	New York	NY	USA	10044	07/26/13	n/a	KM577017	n/a
**JX124**	F	Pam	B2	New York	NY	USA	10044	07/26/13	n/a	KM576934	n/a
**JX125**	F	Pam	B2	New York	NY	USA	10044	07/26/13	n/a	KM576945	n/a
**JX126**	F	Pam	B2	New York	NY	USA	10044	07/26/13	n/a	KM576939	KM591614
**JX127**	F	Pam	B2	New York	NY	USA	10044	07/26/13	n/a	KM577077	n/a
**JX128**	F	Pam	B2	New York	NY	USA	10044	07/26/13	n/a	KM577092	n/a
**JX129**	F	Pam	A3	New York	NY	USA	10065	07/24/13	n/a	KM576935	n/a
**JX130**	F	Pam	A3	New York	NY	USA	10065	07/31/13	n/a	KM577157	KM591649
**JX131**	F	Pam	A3	New York	NY	USA	10024	07/31/13	n/a	KM577051	KM591617
**JX132**	F	Pam	A1	New York	NY	USA	10065	July 2013	n/a	KM576938	n/a
**JX133**	F	Pam	A1	New York	NY	USA	10065	July 2013	n/a	KM576995	n/a
**JX134**	F	Pam	A1	New York	NY	USA	10065	July 2013	n/a	KM577055	n/a
**JX135**	F	Pam	A1	New York	NY	USA	10065	July 2013	n/a	KM576963	n/a
**JX136**	F	Pam	A1	New York	NY	USA	10065	July 2013	n/a	KM576982	n/a
**JX137**	F	Pam	C1	New York	NY	USA	10044	08/01/13	n/a	KM576988	KM591657
**JX138**	F	Sla	n/a	Tempe	AZ	USA	85284	08/13/13	n/a	KM577065	n/a
**JX139**	F	Pam	C1	New York	NY	USA	10065	09/03/13	n/a	KM576976	KM591672
**JX140**	F	Pam	B2	Boston	MA	USA	02115	08/10/13	n/a	KM577093	KM591619
**JX141**	F	Pam	A1	New York	NY	USA	10024	08/06/13	n/a	KM577097	n/a
**JX142**	F	Pam	A3	New York	NY	USA	10065	08/02/13	n/a	KM576966	n/a
**JX143**	F	Slo	n/a	Hilo	HI	USA	96720	08/18/13	n/a	KM577131	n/a
**JX144**	F	Gpo	n/a	Ithaca	NY	USA	14456	08/13/13	n/a	KM577153	n/a

**JX145**	F	Pam	B2	Rowlett	TX	USA	75088	08/12/13	n/a	KM577156	KM591618
**JX146**	F	Pfu	n/a	Newport News	VA	USA	23601	08/16/13	n/a	KM577016	KM591624
**JX147**	F	Pam	A1	New York	NY	USA	10001	08/10/13	n/a	KM577075	n/a
**JX148**	F	Pam	C1	Washington DC	DC	USA	20013	08/14/13	n/a	KM576948	KM591635
**JX149**	F	Pam	A1	Washington DC	DC	USA	20013	08/14/13	n/a	KM577114	n/a
**JX150**	F	Pam	C1	Washington DC	DC	USA	20013	08/16/13	n/a	KM577034	KM591651
**JX151**	F	Pam	C1	Washington DC	DC	USA	20013	08/15/13	n/a	KM577002	KM591622
**JX152**	F	Pam	A1	Washington DC	DC	USA	20013	08/08/13	n/a	KM577019	n/a
**JX153**	F	Pam	B3	Washington DC	DC	USA	20013	07/31/13	n/a	KM577135	KM591680
**JX154**	F	Pam	A1	Washington DC	DC	USA	20013	08/07/13	n/a	KM577060	n/a
**JX155**	F	Pam	C1	Washington DC	DC	USA	20013	07/23/13	n/a	KM576985	n/a
**JX156**	F	Pam	A1	Washington DC	DC	USA	20013	07/19/13	n/a	KM577031	n/a
**JX157**	F	Pam	A3	Buenos Aires	n/a	Argentina	n/a	July 2013	n/a	KM576928	n/a
**JX158**	F	Pam	A3	Buenos Aires	n/a	Argentina	n/a	July 2013	n/a	KM577149	KM591633
**JX159**	F	Pam	A4	Buenos Aires	n/a	Argentina	n/a	July 2013	n/a	KM577152	KM591609
**JX160**	F	Pfu	n/a	Buenos Aires	n/a	Argentina	n/a	July 2013	n/a	KM577133	KM591650
**JX161**	F	Pam	A1	New York	NY	USA	10024	08/10/13	n/a	KM577059	n/a
**JX162**	N	n/a	n/a	New York	NY	USA	10044	09/05/13	n/a	n/a	n/a
**JX163**	F	Pam	C1	New York	NY	USA	10044	08/25/13	n/a	KM577096	KM591628
**JX164**	F	Pam	A3	New York	NY	USA	10065	08/13/13	n/a	KM577074	n/a
**JX165**	F	Pam	C1	New York	NY	USA	10024	08/05/13	n/a	KM576970	n/a
**JX166**	F	Pam	C1	New York	NY	USA	10044	08/30/13	n/a	KM577103	KM591642
**JX167**	F	Pam	A1	New York	NY	USA	10065	08/02/13	n/a	KM576943	n/a
**JX168**	F	Pfu	n/a	Greenville	SC	USA	29609	08/24/13	n/a	KM576972	KM591620
**JX169**	F	Pam	B2	New York	NY	USA	11103	08/28/13	n/a	KM576953	n/a
**JX170**	F	Pam	C1	New York	NY	USA	10024	09/09/13	AMNH_IZC 00114334	KM577082	n/a
**JX171**	F	Pam	C1	New York	NY	USA	10024	09/09/13	AMNH_IZC 00114335	KM577041	n/a
**JX172**	F	Pam	A3	New York	NY	USA	10025	08/14/13	n/a	KM577138	n/a
**JX173**	F	Pam	C1	New York	NY	USA	10024	09/08/13	n/a	KM576967	n/a
**JX174**	F	Pam	A3	New York	NY	USA	10024	08/10/13	n/a	KM576960	n/a
**JX175**	F	Pam	B2	New York	NY	USA	10003	08/01/13	n/a	KM577071	n/a
**JX176**	F	Pam	A3	New York	NY	USA	10022	10/04/13	AMNH_IZC 00114348	KM576983	n/a
**JX177**	F	Pam	A3	New York	NY	USA	10003	10/06/13	n/a	KM577042	n/a
**JX178**	F	Pam	C1	Kea'au	HI	USA	96749	09/29/13	n/a	KM576958	n/a
**JX179**	N	n/a	n/a	Kea'au	HI	USA	96249	09/28/13	n/a	n/a	n/a
**JX180**	F	Pam	A1	New York	NY	USA	10025	09/09/13	n/a	KM577061	n/a
**JX181**	F	Pam	C1	New York	NY	USA	10025	10/11/13	n/a	KM577039	n/a
**JX182**	F	Pam	C1	New York	NY	USA	n/a	10/02/13	n/a	KM576996	n/a
**JX183**	N	n/a	n/a	New Orleans	LA	USA	70118	08/08/13	n/a	n/a	n/a
**JX184**	N	n/a	n/a	New Orleans	LA	USA	70118	09/11/13	n/a	n/a	n/a
**JX185**	F	Pam	A2	New York	NY	USA	10024	09/10/13	n/a	KM577027	n/a
**JX186**	F	Pam	A2	New York	NY	USA	10024	09/10/13	n/a	KM577043	n/a
**JX187**	F	Pam	A2	New York	NY	USA	10024	09/10/13	n/a	KM577056	n/a
**JX188**	F	Pam	C1	New York	NY	USA	10024	09/10/13	AMNH_IZC 00114346	KM577089	n/a
**JX189**	F	Pam	A2	New York	NY	USA	10024	09/10/13	n/a	KM577006	n/a
**JX190**	F	Pam	A3	New York	NY	USA	10024	09/10/13	AMNH_IZC 00114345	KM576991	n/a
**JX191**	F	Pam	B2	New York	NY	USA	10024	09/10/13	n/a	KM577032	n/a
**JX192**	F	Pam	A3	New York	NY	USA	10024	09/10/13	n/a	KM577040	n/a
**JX193**	F	Pam	A3	New York	NY	USA	10024	09/10/13	n/a	KM577052	n/a

**JX194**	F	Pam	B2	New York	NY	USA	10024	09/10/13	AMNH_IZC 00114347	KM577155	n/a
**JX195**	F	Pam	C1	New York	NY	USA	10024	09/10/13	n/a	KM577018	n/a
**JX196**	F	Pam	A2	New York	NY	USA	10024	09/10/13	n/a	KM577009	n/a
**JX197**	F	Pam	B2	New York	NY	USA	10024	09/10/13	AMNH_IZC 00114344	KM577151	n/a
**JX198**	N	n/a	n/a	New York	NY	USA	10024	09/10/13	n/a	n/a	n/a
**JX199**	F	Pam	C1	New York	NY	USA	10024	09/10/13	n/a	KM576949	n/a
**JX200**	F	Pam	A1	New York	NY	USA	10024	09/10/13	n/a	KM576923	n/a
**JX201**	F	Pam	B2	New York	NY	USA	10024	09/10/13	n/a	KM577013	n/a
**JX202**	N	n/a	n/a	New York	NY	USA	10024	09/10/13	n/a	n/a	n/a
**JX203**	N	n/a	n/a	New York	NY	USA	10024	09/10/13	n/a	n/a	n/a
**JX204**	F	Pam	A2	New York	NY	USA	10024	09/10/13	n/a	KM576930	n/a
**JX205**	F	Pam	A2	New York	NY	USA	10024	09/10/13	n/a	KM577073	n/a
**JX206**	N	n/a	n/a	New York	NY	USA	10024	09/10/13	n/a	n/a	n/a
**JX207**	F	Pam	C1	New York	NY	USA	10024	09/24/13	n/a	KM577105	n/a
**JX208**	F	Pam	A1	Washington DC	DC	USA	20013	09/02/13	n/a	KM576984	n/a
**JX209**	F	Pam	C1	Washington DC	DC	USA	20013	08/29/13	n/a	KM577110	n/a
**JX210**	F	Pam	C1	Washington DC	DC	USA	20013	08/24/13	n/a	KM577150	n/a
**JX211**	N	n/a	n/a	Washington DC	DC	USA	20013	09/04/13	n/a	n/a	n/a
**JX212**	F	Pam	C1	Washington DC	DC	USA	20013	08/24/13	n/a	KM577021	n/a
**JX213**	F	Pam	A3	New York	NY	USA	10024	09/06/13	n/a	KM577121	n/a
**JX214**	F	Pam	C1	New York	NY	USA	10024	09/24/13	n/a	KM576971	n/a
**JX215**	N	n/a	n/a	Newport News	VA	USA	23601	09/17/13	n/a	n/a	n/a
**JX216**	N	n/a	n/a	New Orleans	LA	USA	70118	09/03/13	n/a	n/a	n/a
**JX217**	F	Pfu	n/a	Evans	GA	USA	30809	09/08/13	n/a	KM577101	n/a
**JX218***	F	n/a	n/a	n/a	GA	USA	30809	09/15/13	n/a	KM577062	n/a
**JX219**	N	n/a	n/a	Newport News	VA	USA	23601	09/12/13	n/a	n/a	n/a
**JX220**	F	Pfu		Newport News	VA	USA	23601	09/07/13	n/a	KM576933	n/a
**JX221**	N	n/a	n/a	New York	NY	USA	10024	10/15/13	n/a	n/a	n/a
**JX222**	F	Pam	C1	Newport News	VA	USA	23601	09/12/13	n/a	KM577022	n/a
**JX223**	N	n/a	n/a	Wichita	KS	USA	67210	10/07/13	n/a	n/a	n/a
**JX224**	N	n/a	n/a	Wichita	KS	USA	67210	10/07/13	n/a	n/a	n/a
**JX225**	F	Pam	C1	Wichita	KS	USA	67210	10/07/13	n/a	KM577047	n/a
**JX226**	F	Pam	C1	Wichita	KS	USA	67210	10/07/13	n/a	KM577132	n/a
**JX227**	F	Pam	C1	Caracas	n/a	Venezuela	n/a	08/01/13	n/a	KM577112	n/a
**JX228**	F	Pam	C2	Caracas	n/a	Venezuela	n/a	08/01/13	n/a	KM577049	n/a
**JX229**	F	Pam	C1	Caracas	n/a	Venezuela	n/a	08/01/13	n/a	KM577079	n/a
**JX230**	F	Pam	C1	Caracas	n/a	Venezuela	n/a	08/01/13	n/a	KM576994	n/a
**JX231**	F	Pam	C1	Caracas	n/a	Venezuela	n/a	08/01/13	n/a	KM577108	n/a
**JX232**	F	Pam	C1	Caracas	n/a	Venezuela	n/a	08/01/13	n/a	KM577044	n/a
**JX233**	F	Pam	C1	Caracas	n/a	Venezuela	n/a	08/01/13	n/a	KM577148	n/a
**JX234**	F	Pam	C1	Caracas	n/a	Venezuela	n/a	08/01/13	n/a	KM576920	n/a
**JX235**	F	Pam	C1	Caracas	n/a	Venezuela	n/a	08/01/13	n/a	KM577140	n/a
**JX236**	F	Pam	A3	Caracas	n/a	Venezuela	n/a	08/01/13	n/a	KM576937	n/a
**JX237**	F	Pam	C1	Caracas	n/a	Venezuela	n/a	09/01/13	n/a	KM577057	n/a
**JX238**	N	n/a	n/a	Caracas	n/a	Venezuela	n/a	09/01/13	n/a	n/a	n/a
**JX239**	F	Pam	C1	Caracas	n/a	Venezuela	n/a	09/01/13	n/a	KM577125	n/a
**JX240****	F	n/a	n/a	Caracas	n/a	n/a	n/a	n/a	n/a	KM577141	n/a
**JX241**	F	Pam	C1	Caracas	n/a	Venezuela	n/a	09/01/13	n/a	KM577067	n/a
**JX242**	N	n/a	n/a	Caracas	n/a	Venezuela	n/a	09/01/13	n/a	n/a	n/a

**JX243**	N	n/a	n/a	New York	NY	USA	10460	01/11/13	n/a	n/a	n/a
**JX244**	N	n/a	n/a	New York	NY	USA	10460	01/11/13	n/a	n/a	n/a
**JX245**	F	Pam	B2	New York	NY	USA	10460	01/11/13	n/a	KM577012	n/a
**JX246**	N	n/a	n/a	New York	NY	USA	10460	01/11/13	n/a	n/a	n/a
**JX247**	N	n/a	n/a	New York	NY	USA	10460	01/11/13	n/a	n/a	n/a
**JX248**	F	Pam	B2	New Orleans	LA	USA	70118	12/04/13	n/a	KM577083	n/a
**JX249**	F	Pam	B2	Arecibo	PR	USA	00612	12/08/13	n/a	KM577008	n/a
**JX250**	F	Pam	C1	New York	NY	USA	10024	11/12/13	n/a	KM577064	n/a
**JX251**	F	Pfu	n/a	Raleigh	NC	USA	27607	11/03/13	n/a	KM577144	n/a
**JX252**	F	Pam	A3	Brisbane	QLD	Australia	n/a	10/27/13	n/a	KM577007	n/a
**JX253**	N	n/a	n/a	Newport News	VA	USA	23608	11/13/13	n/a	n/a	n/a
**JX254**	N	n/a	n/a	New York	NY	USA	10016	10/07/13	n/a	n/a	n/a
**JX255**	N	n/a	n/a	New York	NY	USA	10009	10/08/13	n/a	n/a	n/a
**JX256**	F	Pfu	n/a	Tuscaloosa	AL	USA	35475	10/16/13	n/a	KM577142	n/a
**JX257**	F	Pam	C1	Washington DC	DC	USA	20037	n/a	n/a	KM576989	n/a
**JX258**	F	Pam	C1	New York	NY	USA	11355	July 2013	n/a	KM576981	n/a
**JX259**	F	Pam	B4	Philadelphia	PA	USA	19131	09/01/13	n/a	KM577036	n/a
**JX260**	F	Pam	B1	New York	NY	USA	10065	10/30/13	n/a	KM576956	n/a
**JX261**	F	Pam	A3	New York	NY	USA	10024	10/28/13	n/a	KM577068	n/a
**JX262**	F	Pam	A3	Newark	NJ	USA	07102	01/10/14	n/a	KM576926	n/a
**JX263**	F	Pam	A3	New York	NY	USA	10024	n/a	n/a	KM577045	n/a
**JX264**	F	Pam	A1	New York	NY	USA	10065	12/01/13	n/a	KM577037	n/a
**JX265**	N	n/a	n/a	Columbus	OH	USA	43212	12/09/13	n/a	n/a	n/a
**JX266**	F	Pam	C1	Kailua-Kona	HI	USA	96740	12/20/13	n/a	KM576987	n/a
**JX267**	F	Pam	A1	Belize City	n/a	Belize	n/a	12/26/13	n/a	KM577005	n/a
**JX268**	F	Slo	n/a	Rupununi Savannah	n/a	Guyana	n/a	06/13/13	n/a	KM577124	n/a
**JX269**	F	Pam	B5	Rupununi Savannah	n/a	Guyana	n/a	06/13/13	n/a	KM576940	n/a
**JX270**	N	n/a	n/a	Rupununi Savannah	n/a	Guyana	n/a	06/13/13	n/a	n/a	n/a
**JX271**	F	Pam	A2	Rupununi Savannah	n/a	Guyana	n/a	06/17/13	n/a	KM577126	n/a
**JX272**	F	Pam	B5	Rupununi Savannah	n/a	Guyana	n/a	06/13/13	n/a	KM577080	n/a
**JX273**	F	Pam	A3	Newark	NJ	USA	07172	Oct 2013	n/a	KM577102	n/a
**JX274**	F	Pam	A3	Newark	NJ	USA	07172	Oct 2013	n/a	KM577069	n/a
**JX275**	N	n/a	n/a	Madewini	n/a	Guyana	n/a	08/20/13	n/a	n/a	n/a
**JX276**	F	Pam	B4	Philadelphia	PA	USA	19131	11/01/13	n/a	KM577001	n/a
**JX277**	F	Pam	B4	Philadelphia	PA	USA	19131	11/01/13	n/a	KM577147	n/a
**JX278**	F	Pam	B4	Philadelphia	PA	USA	19131	11/01/13	n/a	KM576957	n/a
**JX279**	N	n/a	n/a	Philadelphia	PA	USA	19131	07/01/13	n/a	n/a	n/a
**JX280**	F	Pam	C1	New York	NY	USA	10024	01/10/14	n/a	KM577095	n/a
**JX281**	F	Pam	A3	New York	NY	USA	10027	12/31/13	n/a	KM577020	n/a
**JX282**	F	Pam	A3	New York	NY	USA	10027	10/15/13	n/a	KM576959	n/a
**JX283**	F	Pam	A3	Columbus	OH	USA	43212	12/18/13	n/a	KM576955	n/a
**JX284**	N	n/a	n/a	New York	NY	USA	10028	12/21/13	n/a	n/a	n/a

Notes:

* Best match GenBank 94% to Pfu record JQ350729; best match Barcode of Life Datasystems (BOLD) 99% to unidentified roach collected in Texas, sample ID BIOUG2490-D05.

** Best match GenBank 100% to Bge record HM996892, but distant from other Bge records; best match BOLD 97% to multiple unpublished *Pycnocelus surinamensis* records.

Table S2. Previous GenBank records of *P. americana COI* barcodes included in the analyses (N=24) and of other cockroaches used as reference sequences for species identifications. Hyphens indicate missing data. Abbreviation: n/a = not applicable.

GenBank Accession No.	Species name	City, State	Country	Haplogroup
**AM114927**	*Periplaneta americana*	-	-	A3
**GU947663**	*Periplaneta americana*	-	China	B2
**AY165646**	*Periplaneta americana*	-	-	A6
**JN900479**	*Periplaneta americana*	-	Iran	B1
**JQ267476**	*Periplaneta americana*	Kashan	Iran	B1
**JQ267477**	*Periplaneta americana*	Kashan	Iran	B1
**JQ267478**	*Periplaneta americana*	Kashan	Iran	B1
**JQ267479**	*Periplaneta americana*	Tehran	Iran	B1
**JQ267480**	*Periplaneta americana*	Tabriz	Iran	B1
**JQ267481**	*Periplaneta americana*	Tabriz	Iran	B1
**JQ267482**	*Periplaneta americana*	Tehran	Iran	B1
**JQ267483**	*Periplaneta americana*	Tehran	Iran	B1
**JQ267484**	*Periplaneta americana*	Isfahan	Iran	B1
**JQ267485**	*Periplaneta americana*	Tehran	Iran	A3
**JQ267486**	*Periplaneta americana*	Tehran	Iran	A1
**JQ267487**	*Periplaneta americana*	Tehran	Iran	A3
**JQ267488**	*Periplaneta americana*	Tehran	Iran	B4
**JQ267489**	*Periplaneta americana*	Tehran	Iran	B4
**JQ350707**	*Periplaneta americana*	-	Korea	B7
**JX402724**	*Periplaneta americana*	Gainesville, Florida	USA	C3
**KC617844**	*Periplaneta americana*	-	USA	C1
**KC617845**	*Periplaneta americana*	-	USA	A1
**KC617846**	*Periplaneta americana*	Indiana	USA	A1
**KF640070**	*Periplaneta americana*	-	China	B6
**AM114928**	*Periplaneta australasiae*	-	-	n/a
**KF640069**	*Periplaneta australasiae*	-	China	n/a
**AM114930**	*Periplaneta brunnea*	-	-	n/a

**JQ350729**	*Periplaneta fuliginosa*	-	Korea	n/a
**AB126004**	*Periplaneta fuliginosa*	-	-	n/a
**AM114929**	*Periplaneta japonica*	-	-	n/a
**JQ350708**	*Periplaneta japonica*	-	Korea	n/a
**KC407710**	*Periplaneta japonica*	-	Korea	n/a
**KC407711**	*Periplaneta japonica*	-	Korea	n/a
**KF372519**	*Gromphadorhina portentosa*	-	-	n/a
**EU253834**	*Supella longipalpa*	-	-	n/a
**JQ267494**	*Shellfordella lateralis*	-	-	n/a

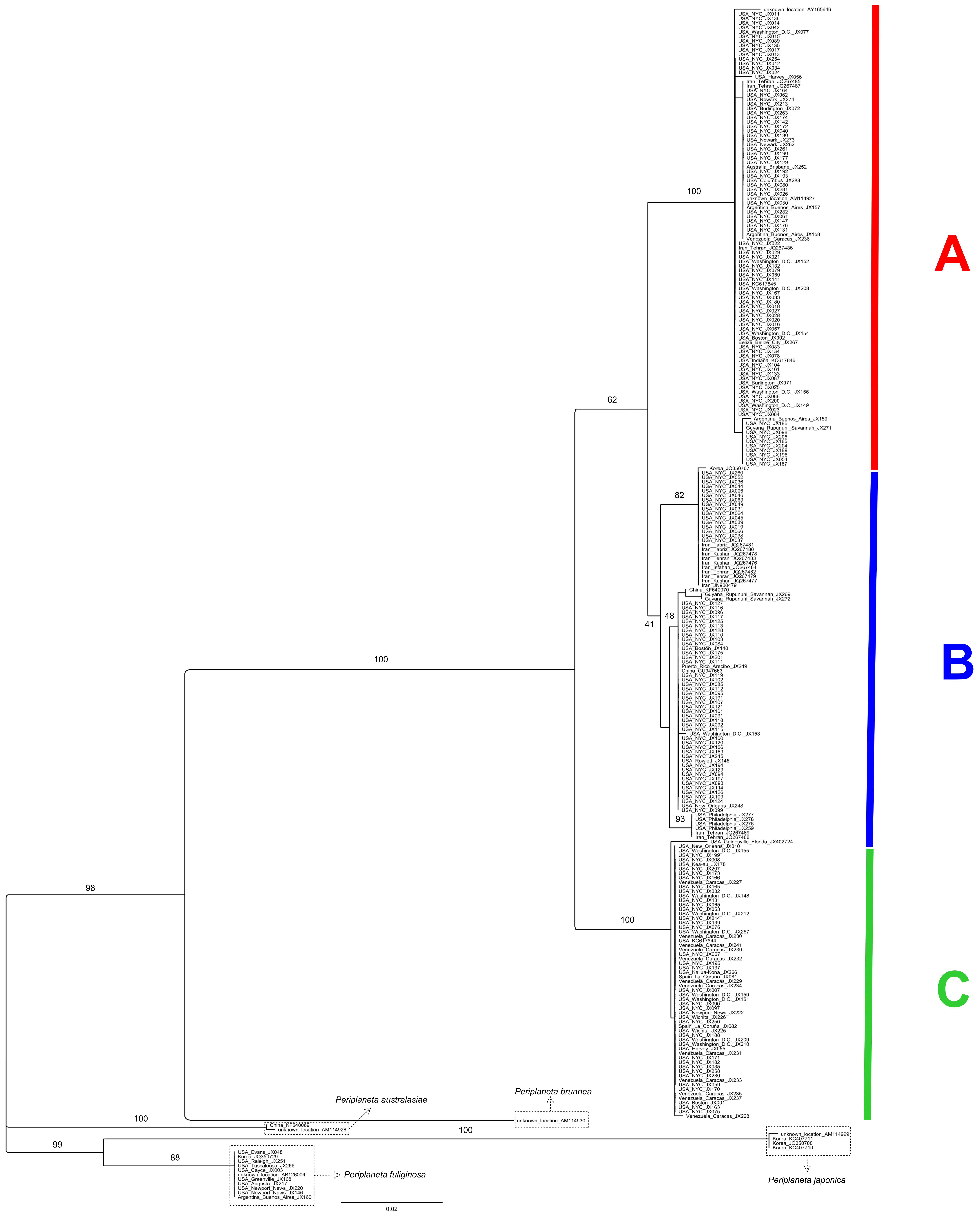

Figure S1. Randomized Axelerated Maximum Likelihood (RAxML) tree for mitochondrial *COI* sequences of *Periplaneta americana*, *P. brunnea*, *P. australasiae*, *P. japonica* and *P. fuliginosa*. Colors depict *P. americana COI* haplogroups A, B, and C. Bootstrap support values (1,000 replications) are shown at branches. Samples taken from GenBank are recognizable by their accession number.
